# Freezing and Thawing Human Embryonic Stem Cells

**DOI:** 10.3791/1555

**Published:** 2009-12-24

**Authors:** Lia Kent

**Affiliations:** Research and Development, Stemgent

## Abstract

Since James Thomson *et al* developed a technique in 1998 to isolate and grow hES in culture, freezing cells for later use and thawing and expanding cells from a frozen stock have become important procedures performed in routine hES cell culture. Since hES cells are very sensitive to the stresses of freezing and thawing, special care must taken. Here we demonstrate the proper technique for rapidly thawing hES cells from liquid nitrogen stocks, plating them on mouse embryonic feeder cells, and slowly freezing them for long-term storage.

**Figure:**
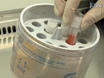


## Protocol

### 1. Thawing hES cells

#### Pre-experimental Set-up

1. One day prior to thawing hES cells, plate a feeder layer of irradiated mouse embryonic fibroblasts (MEF), CF1 strain, on one well of a gelatin-coated 6-well tissue culture plate in MEF culture medium (D-MEM basal medium supplemented with 10% heat-inactivated FBS and 1% non-essential amino acids). Incubate overnight at 37°C and 5% CO_2_.
2. Before removing the hES cells from liquid nitrogen, be sure to have all necessary equipment and reagents in place and ready to use to ensure an efficient and successful thaw.

#### Thawing the hES Cells

1. Remove a cryogenic vial of hES cells from the liquid nitrogen storage tank using metal forceps.
2. Roll the vial between gloved hands for 3-5 seconds to remove the frost.
3. Record the information on the label of the vial.
4. Using metal forceps, immerse the vial into a 37°C water bath. Swirl the vial gently and observe the progress of the thaw often (but quickly!) by holding the vial up to the light to see the size of the ice crystal. Do not submerge the cap of the vial in the water bath as this could contaminate the cells.
5. When only a small ice crystal remains, submerge the entire vial in ethanol to sterilize.
6. In a sterile biological safety cabinet, transfer the contents of the cryogenic vial directly to the bottom of a 15 mL conical tube.
7. Slowly add 4 mL of hES cell culture medium (D-MEM/F-12 basal medium supplemented with 20% Knockout serum replacement, 1% non-essential amino acids, 1% L-glutamine, 0.2% β- mercaptoethanol and 4 ng/mL bFGF) to the tube. Gently rock to continually mix the cells as the new medium is added to the tube.
8. Centrifuge the cells for 5 minutes at 200 x g.

#### Plating the hES Cells

1. While the hES cells are in the centrifuge, prepare the MEF feeder plate by labeling with the appropriate hES cell information: cell line, passage number, and thaw date.
2. When there is approximately 2 minutes left on the spin time, aspirate the MEF culture medium and add 1.0 mL of PBS to the well.
3. Bring the pelleted hES cells back to the biological safety cabinet, and carefully aspirate the supernatant. Be careful not to aspirate the cell pellet, but remove as much supernatant as possible, as this solution contains DMSO from the freezing medium.
4. Re-suspend the pellet very gently by adding 2.5 mL of hES cell culture medium using a 5 mL glass pipet and pipetting 3-4 times.
5. Aspirate the PBS from the MEF feeder well and slowly add all 2.5 mL of the hES cell suspension to the prepared well of the 6-well plate.
6. Place the plate into the 37°C incubator and carefully slide the plate forward to back, and side to side to evenly distribute the cells throughout the well. Do not swirl the plate in a circular pattern to avoid concentrating the colonies in the center.
7. Allow the cells to attach at 37°C and 5% CO_2_ overnight.
8. The day after the thaw, remove the medium (with any floating cells) and add 2.5 ml of fresh hES cell culture medium to the well.
9. Optional: The removed medium and cells can be transferred to a separate prepared MEF feeder plate. This step often generates new colonies that can be cultured in parallel with cells from the original thaw.
10. Incubate the plates at 37°C and 5% CO_2_ overnight.

### 2. Freezing the hES Cells

1. While the hES cells are in the centrifuge, label the cryogenic vials inside the biological safety cabinet, including the cell line, passage number, and freezing date. The typical density for freezing hES cells is one well of cells (from a 6-well plate) per cryogenic vial.
2. When the hES cells are finished centrifuging, bring the tubes back to the biological safety cabinet. Aspirate the supernatant from the tube, being careful not to disturb the loosely-packed cell pellet.
3. Resuspend the cell pellet with the appropriate amount of hES cell culture medium: 0.5 mL hES cell culture medium per cryogenic vial. Be very gentle when pipetting, as the hES cells recover better when frozen in relatively large colony pieces.
4. Slowly, while gently shaking the tube, add the appropriate amount (0.5 mL per cryovial) of 2X hES cell freezing medium (60% defined FBS, 20% hES cell culture medium, and 20% DMSO) to the cells. Once the freezing medium is added, pipet the solution extremely gently one to two times to mix.
5. Quickly but gently, add 1 mL of the cell suspension to each cryogenic vial.
6. Transfer the vials into an isopropanol freezing container and place in a -80°C freezer overnight. The cells will freeze at 1°C per minute in the isopropanol freezing container.
7. The following day, quickly transfer the frozen vials to a liquid nitrogen storage tank using metal forceps.

### Representative Results

The first day after human ES cells are thawed, small colonies may appear transparent and may be difficult to see under the microscope. Since newly thawed ES cells tend to proliferate slowly, they may take a few days to appear as established colonies (Figure 1).

**Figure 1. Cell recovery and growth. **hES cells thawed from liquid nitrogen were imaged**** 1, 7 and 11 days after thawing.

## Discussion

The accompanying video demonstrates a method for thawing and freezing hES cells. Cells frozen in liquid nitrogen should be thawed *bath as quickly as possible* to obtain the best possible recovery. Remember to be extremely careful when pipetting thawing cells: minimize handling of the cells and pipette gently. One vial of hES cells can be plated onto either one well of a 6-well plate, or all wells of a 4-well plate and immediately placed in the incubator. Since culturing in four separate wells reduces the possibility of losing the entire culture to contamination, and makes it easier to locate hES cell colonies on the smaller surface area, the 4-well plate is recommended when thawing hES cells for the first time.

The first day after thawing hES cells, colonies are small and may be transparent. Replenish the hES cell culture medium every day, even if the colonies are not apparent under the microscope since it may take a few days in culture for hES cells to appear as established colonies. It is important to use a freshly plated MEF feeder layer when thawing cells, as the colonies often do not reach an appropriate size for passaging until 10   12 days after the thaw.

When thawing a *low passage* vial of hES cells, it is good practice to expand and freeze additional vials to maintain a stock of low passage cells for future culture and experiments. It is also a good idea to routinely freeze all "extra" healthy hES cells to maintain frozen stocks. The most successful thaws and subsequent cultures were frozen as high-quality, undifferentiated, actively dividing hES cell colonies. hES cells recover from a freeze more efficiently if handled gently as larger cell aggregates. The final aggregate size should be similar to (or slightly larger than) the size of aggregates plated after a routine passage. 
